# Fatigue, cognitive complaints, dyspnea, anxiety, and depression as post-COVID syndrome: a cross-sectional study in Sfax, Southern Tunisia

**DOI:** 10.11604/pamj.2025.52.3.45439

**Published:** 2025-09-01

**Authors:** Zeineb Mallek, Rahma Gargouri, Hanen Maamri, Maissa Ben Jmeaa, Mouna Baklouti, Mohamed Amine Hadj Sassi, Eya Ayadi, Feiza Kallel, Najla Bahloul, Rim Khmekhem, Nessrine Kallel, Jihen Jedidi, Imen Sboui, Yosra Mejdoub, Nedia Moussa, Sourour Yaich, Sami Kammoun

**Affiliations:** 1 Community Health and Epidemiology Department, Hedi Chaker University Hospital, University of Sfax, Sfax, Tunisia,; 2 Pneumology Department, Hedi Chaker Hospital, Sfax, Tunisia,; 3 High School of Health Science and Technics of Sfax, Sfax, Tunisia

**Keywords:** COVID-19, public health, dyspnea, fatigue, cognition, anxiety, depression

## Abstract

**Introduction:**

post-COVID refers to symptoms and health problems that continue or develop after the initial phase of a COVID-19 infection has resolved. It represents a huge public health issue, leading to considerable illness and lowering the quality of life for those affected. Our study aimed first to provide a general description of post-COVID conditions and then to examine the specific aspects of fatigue, cognitive complaints, dyspnea, anxiety, and depression in patients followed up in the pulmonology department of CHU Hedi Chaker, Sfax.

**Methods:**

we conducted a cross-sectional study in the pulmonology department of CHU Hedi Chaker, Sfax in 2024 using an anonymous self-questionnaire with 4 validated scales to assess cognitive complaint, fatigue, dyspnea, as well as anxiety, and depression.

**Results:**

overall, 75 participants were included, with a sex ratio of 0.63 and a median age of 45 years (interquartile range (IQR= [35-66 years]). The evaluation of the impact of COVID-19 symptoms on daily life showed that 45.3% of the participants (n= 34) reported that fatigue had a severe impact on daily life, while 33.3% (n= 25) reported severe impacts from stress and negative emotions. The prevalences of fatigue, cognitive complaints, very severe dyspnea, anxiety, and depression based on the scale used were 88% (n= 66), 80% (n= 60), 14.7% (n= 11), 44% (n= 33) and 28.6% (n= 21), respectively. The factor most associated with fatigue in the population was cognitive complaints (OR= 25.3, p<0.001), while the factor most associated with dyspnea was age between 70 and 95 (OR= 12.85; p= 0.006). Furthermore, the factor most significantly associated with cognitive complaints was anxiety with an OR of 25.3 and p<0.001. As for anxiety and depression, the most associated factors were cognitive complaints and depression (OR=25.3; p<0.001) vs (OR= 27.1; p<0.001), respectively.

**Conclusion:**

our study shows significant prevalences of post-COVID symptoms, such as fatigue, cognitive complaints, severe dyspnea, anxiety, and depression. Thus, post-COVID syndrome poses a significant public health challenge due to its wide array of symptoms that continue to affect individuals' health.

## Introduction

COVID-19 caused by SARS-CoV-2, emerged in late 2019 in Wuhan, China. It swiftly escalated into a global pandemic that has presented significant challenges to healthcare systems and societies worldwide. COVID-19 ranked as the third highest cause of mortality globally in 2020 and moved to number two by 2021 [1]. The World Health Organization (WHO) estimates that COVID-19 causes over 1,700 deaths globally per week as of July 2024 [2]. Tunisia, too, has felt the impact of this pandemic, recording 1,156,613 cases and 29,494 deaths as of October 25^th^ 2023 [3]. Despite being predominantly recognized as a respiratory infection, COVID-19 can impact almost every organ and system in the body. Frequent signs and symptoms include chills, fever, and sore throat [4]. Numerous side effects have been reported in the literature, including cardiac failure, arrhythmias, and myocardial damage [5]. Among hospitalized COVID-19 patients, especially those in Intensive Care Units, psychiatric problems like depression, impulsivity, acute stress disorder, insomnia, and post-traumatic stress disorder are also common [6]. Furthermore, there has been evidence of neurological problems linked to COVID-19, including headaches, dizziness, altered consciousness, acute ischemic strokes, and intracranial hemorrhages [7]. Many survivors of SARS-CoV-2 infection experience symptoms that persist beyond the initial recovery period, commonly known as post-COVID symptoms. According to the definition provided by the WHO, the post-COVID-19 condition, widely known as long-term COVID-19 is defined as the continuation or development of new symptoms 3 months after the initial SARS-CoV-2 infection, with these symptoms lasting for at least 2 months with no other explanation [8].

An estimated 17 million people in the WHO European Region experienced long-term effects from COVID-19 between 2020 and 2021. It has been reported in at least 10% of patients recovering from SARS-CoV-2 infections and possibly up to 50% to 70% of hospitalized cases [9]. Despite the possible reduction in major outbreaks, the risk of developing persistent symptoms after COVID-19 remains significant, affecting 10-20% of those infected [10]. Long COVID comprises a diverse set of symptoms, with over 200 identified to date, varying in severity and evolving. Common symptoms that significantly impact daily life include persistent fatigue, post-exertional malaise, memory impairment, difficulty concentrating, and shortness of breath [10]. Quality of life was reduced by 51% to 62% after three to six months among COVID-19 survivors [11]. After several months, negative changes in mental health, such as increased anxiety, depression, and post-traumatic stress disorder, were often reported symptoms [11]. In this regard, our study aimed to first provide a general description of post-COVID conditions and then to determine the prevalence and associated factors of specific symptoms, including fatigue, cognitive complaints, dyspnea, anxiety, and depression, in patients followed up at the pulmonology department of CHU Hedi Chaker, Sfax.

## Methods

**Study design and settings:** a cross-sectional study was conducted using an anonymous questionnaire, including patients with post-COVID-19 syndrome followed at the CHU Hedi Chaker pneumology department in Sfax, Southern Tunisia, during the period from 28^th^ January 2024 to 10^th^ March 2024.

**Inclusion criteria:** we included patients aged 18 or older with post-COVID-19 syndrome, free of cognitive impairment and previous psychiatric pathology, after obtaining written informed consent from the patients.

**Non inclusion criteria:** those who presented a sign suggestive of a new COVID-19 infection at the time of the study, or who had a history of cognitive impairment or psychiatric disorders, and who did not agree to participate in this study were excluded.

**Exclusion criteria:** incomplete or doubtful answers were eliminated. The minimum sample size was calculated using the following formula:


$$N=\frac{1.96^{2}P0\left(1-P0\right)}{i^{2}}$$

To determine the prevalence of the variable of interest, P0, we relied on the findings from a study conducted in 2021 in Spain, which found a P0 of severe dyspnea equal to 4.4% [12]. Therefore, the number of subjects required was equal to 65 with a precision of 5%.

**Data collection, study instruments, and case definition:** data for this study were collected using an anonymous self-administered questionnaire. It included four parts: the first part focused on the socio-demographic characteristics of the study population such as age, sex, marital status, geographical origin, profession, medical history, and lifestyle habits (smoking, alcohol consumption, and regular physical activity). The second part focused on the clinical features of the acute COVID-19 episode (respiratory and extra-respiratory symptoms, need for hospitalization, and whether or not patients were put on oxygen therapy and/or mechanical ventilation), and vaccination against COVID-19. The third part revolved around the impact of persistent symptoms on patients' daily lives, activities, and functioning, using a Likert scale from 0 (no impact) to 3 (severe impact). The level of perceived overall health was based on two visual analog scales graduated from 1 to 10 points, and the participant had to choose the level of his overall health according to his perception. The last part included four validated scales to assess cognitive complaints, fatigue, dyspnea, anxiety, and depression:

**Assessment of Cognitive complaints:** the Subjective Cognitive Complaints (SCC) was developed in 2004. It included ten questions. The patient was asked to answer “yes” if the item was true or “no” if it was false based on the last six months. Each affirmative answer is scored 1 point. Thus, the score ranges from 0 (no complaint) to 10 (severe complaint). The score was considered abnormal according to the following criteria [13]: A total score greater than or equal to 3; and/or only 1 yes answer to question 5 (forgetting an event); and/or 2 single yes answers to questions B (memory functioning less than subjects of the same age), 4 (orientation), 7 (reduction in activities), or 8 (change in character).

**Assessment of fatigue:** the validated Chalder Fatigue Scale (CFQ-11) was used to assess the fatigue. It contains 11 items: seven related to physical fatigue and four to psychological fatigue. Answers were measured using a Likert scale from 0 (Less than usual) to 3 (Much more than usual) [14]. CFQ-11 also featured a bimodal scoring algorithm where each item response was dichotomized : 0 (0 to 1) out of a total of 0 to 11. By convention, fatigue status was classified using this bimodal scale with a threshold of < 4 vs. = 4 [14].

**Assessment of dyspnea:** dyspnea was measured using a modified scale entitled Modified Medical Research Council Dyspnea (mMRC). This was a unidimensional scale that had 5 rating levels, ranging from 0 (no dyspnea / only for sustained exertion) to 4 (dyspnea at rest / too breathless to go out to leave the house), based on the degrees of the various physical activities precipitating dyspnea [15].

**Assessment of anxiety and depression:** signs of anxiety and depression were assessed using the French version of the Hospital Anxiety and Depression (HAD) scale, a 14-item structured self-questionnaire. A score less than or equal to 7 signifies the absence of symptomatology, between 8 and 10 means doubtful symptomatology (borderline case), and a score greater than or equal to 11 means certain symptomatology [16].

**Ethical considerations:** prior to commencing the study, ethical clearance was sought from the head of the pneumology department. Measures have been taken to respect the rights and freedom of participants. Participants were informed of the purpose of the study, the terms, and conditions of participation using a consent form. The confidentiality of the data obtained and the anonymity of the participants were respected.

**Statistical analysis:** data management and analysis were performed using SPSS 26 version. The results of the continuous variables were presented according to the normality of the variable distribution as mean ± standard deviation or median and interquartile range (IQR). The association between two qualitative variables was made by the Pearson “Chi2” test when the conditions were verified otherwise the exact Fisher test was a remedy. A p lower than 0.05 was considered statistically significant.

## Results

Data had been collected from 83 patients having post-COVID syndrome, of which 75 completed the questionnaire, and were retained for the analysis, giving a response rate of 90.4%. There were 46 females (61.3%) with a sex ratio of 0.63. The median age was 45 years (IQR= [35-66]). Thirty-eight (50.7%) participants had an age group between 18-45 years. According to their geographical origin, 68% (n= 51) came from an urban area. Forty-eight (64%) had a medical history. Regarding their lifestyle habits, 30.7% (n=23) were smokers, 16% (n=12) were alcohol consumers, and 20% (n=15) took part in regular physical activities. When asked about the acute episode of COVID-19, 40% (n= 30) of the participants reported being hospitalized due to COVID-19, 16% (n= 12) had recourse to Oxygen therapy, and 20% (n= 15) were mechanically ventilated. Sixty-eight (90.7%) participants were vaccinated against COVID-19 at the time of the survey (Table 1). The evaluation of the impact of COVID-19 symptoms on daily life showed that 45.3%of the participants (n= 34) reported that fatigue had a severe impact on daily life, and 25 participants (33.3%) reported that stress and negative emotions had a severe impact.

**Table 1:** socio-demographic characteristics, lifestyle habits, and data related to the COVID-19 episode

Characteristics	Number (N)	Percentage (%)
Gender		
Male	29	38.7
Female	46	61.3
**Age group**		
18-45 years	38	50.7
46-69 years	25	33.3
70-95 years	12	16
**Marital status**		
Single	14	18.7
Married	52	69.3
Divorced	2	2.7
Widowed	7	9.3
**Educational level**		
Illiterate	15	20
Primary	7	9.3
Secondary	31	41.3
University	22	29.3
Worker	47	49.3
**Geographical origin**		
Urban	51	68
Rural	24	32
Medical history	48	64
Diabetes	17	22.7
High blood pressure	28	37.3
Asthma	24	24
**Hospitalization due to COVID-19**	30	40
Oxygenation	12	16
Mechanical ventilation	15	20
Vaccination against COVID-19	68	90.7
Tobacco use	23	30.7
Alcohol consumption	12	16
Sport activities	15	20

### Fatigue, cognitive complaints, dyspnea, anxiety and depression

**Average/Median scores:** the median CFQ-11 score for all participants was 20.8±4.1, with a median physical fatigue score of 13.2±3.8 and a median psychological fatigue score of 7.6±2.9. Besides, the median score of cognitive complaints was 7 (IQR = [4-8]). Moreover, the calculation of the HAD score showed a mean score of anxiety and depression equal to 9.3±4.1 and 8.2±3.9, respectively.

**Prevalences** (Table 2): the prevalences of fatigue, cognitive complaints, very severe dyspnea, anxiety, and depression based on the scales used were 88% (n= 66), 80% (n= 60), 14.7% (n= 11), 44% (n= 33) and 28.6% (n= 21), respectively.

**Table 2:** prevalence and factors associated with fatigue, very severe dyspnea, cognitive complaints, certain anxiety and depression

Variable	Fatigue N (%)	OR [95%CI]	Very severe dyspnea N (%)	OR [95%CI]	Cognitive complaints N (%)	OR (95%CI)	Certain anxiety N (%)	OR [95%CI]	Certain depression N (%)	OR [95%CI]
**Prevalence**	66 (88)	-	11 (14.7)	-	60 (80)	-	33 (44)	-	21 (28)	-
**Gender**										
**Male**	24 (82.8)		3 (10.3)		23 (79.3)		13 (44.8)	1.05[0.41-2.7]	9 (31)	1.28[0.45-3.57]
**Female**	42 (91.3)	2.18 [0.53-8.93]	8 (17.4)	1.82[0.44-7.53]	37 (80.4)	1.07[0.34-3.41]	20 (43.5)		12 (26.1)	
**p**	0.29		0.51		0.9		0.9		0.79	
**Worker**										
**Yes**	34 (89.5)		2 (5.4)		29 (78.4)		13 (35.1)		6 (16.2)	
**No**	32(86.5)	1.33[0.32-5.37]	9 (23.7)	5.34[1.08-2.7]	31 (81.6)	1.22[0.39-3.8]	20 (52.6)	2.04[0.81-5.18]	15 (39.5)	3.36[1.13-10]
**p**	0.74		0.04		0.73		0.16		0.025	
**Age Group**										
**18-45 years**	34 (89.5)	1	2 (5.3)	1	32 (84.2)	1	16 (42.1)	1	7 (18.4)	1
**46-69 years**	23 (92)	1.35 [0.23-8]	4 (16)	3.4[0.57-20.34]	20 (80)	1.33 [0.35-4.95]	11 (44)	1.08[0.39-2.99]	8 (32)	2.08[0.64-16.74]
**70-95 years**	9 (75)	0.58[0.09-3.69]	5 (41.7)	12.85[2.07-80.05]	8 (66.7)	2.66[0.6-11.76]	6 (50)	1.37[0.37-5.06]	6 (50)	4.42[1.09-17.91]
**p**	0.35		0.006		0.39		0.89		0.09	
**Cough**										
**Yes**	54 (94.7)	9 [1.96-41.17]	9 (15.8)	1.5[0.29-7.68]	50 (87.7)	5.7[1.68-19.36]	28 (49.1)	2.5[0.79-7.96]	17 (29.8)	1.48[0.43-5.18]
**No**	12 (66.7)		2 (11.1)		10 (55.6)		5 (27.8)		4 (22.2)	
**p**	0.005		1		0.006		0.11		0.53	
**Asthma**										
**Yes**	18 (94.7)	3 [0.9-1.32]	8 (44.4)	14.4[3.25-63.81]	16 (88.9)	2.36[0.48-11.65]	10 (55.6)	1.84[0.63-5.38]	6 (33.3)	1.4[0.45-4.39]
**No**	48 (85.7)		3 (5.3)		44 (77.2)		23 (40.4)		15 (26.3)	
**p**	0.43		**<0.001**		0.49		0.26		0.56	
**Origin**										
**Rural**	23 (95.8)	4.28[0.5-36.35]	7 (29.2)	4.83[1.26-18.62]	21 (87.5)	2.15[0.54-8.49]	11 (45.8)	1.11[0.42-2.95]	10 (41.7)	2.59[0.91-7.42]
**Urban**	43 (84.3)		4 (7.8)		39 (76.5)		22 (43.1)		11 (21.6)	
**p**	0.26		**0.03**		0.36		0.82		0.07	

**Associated factors** (Table 2.1): the determinants of fatigue in our study population were a pathological cognitive complaint score, definite anxiety, and having respiratory symptoms during the acute phase (96.7% vs 53.3%; OR= 25.37; p<0.001), (97.1% vs 80.5%; OR= 8; p= 0.035) and (94.7% vs 66.7%; OR= 9; p= 0.005) respectively. Regarding the factors influencing cognitive complaints, these were observed more frequently among individuals with certain anxiety (96.9% vs 59.5%; OR= 25.37; p<0.001), those who required mechanical ventilation (88.2% vs 62.5%; OR= 4.54; p= 0.014), and those who experienced respiratory symptoms during the acute phase of COVID-19 (87.7% vs 55.6%; OR= 5.7; p= 0.006). Severe dyspnea was found more frequently in non-workers, (23.7% vs 5.4%; OR= 5.34; p= 0.04), in those aged between 70 and 95 years in comparison to those aged between 18 and 45 years (41.7% vs 5.3%; OR= 12.85; p= 0.006), those with asthma (44.4% vs 5.3%; OR= 14.4; p<0.001), those who were under mechanical ventilation (33.3% vs 1%; OR= 4.5; p= 0.04), and those who had a certain depression (33.3% vs 7.4%; OR=6.25; p=0.009). Certain anxiety was more frequent in those who were under mechanical ventilation (66.7% vs 38.3%; OR= 3.21; p= 0.04), those who suffered from certain depression (90.5% vs 25.9%; OR=27.4; p<0.001), those who had a pathological cognitive complaint score (96.9% vs 2.3%; OR= 25.37; p<0.001), and those who suffered from fatigue (96.9% vs 2.3%; OR= 8; p= 0.004). In addition, depression was found more frequently in non-workers (39.5% vs 16.2%; OR=3.36; p= 0.025), those who had very severe dyspnea (63.6% vs 21.9%; OR= 6.25; p= 0.009), and those who suffered from certain anxiety (57.6% vs 4.8%; OR= 27.14; p<0.001).

**Table 2.1:** prevalence and factors associated with fatigue, very severe dyspnea, cognitive complaints, certain anxiety and depression

Variable	Fatigue N (%)	OR [95%CI]	Very severe dyspnea N (%)	OR [95%CI]	Cognitive complaints N (%)	OR (95%CI)	Certain anxiety N (%)	OR [95%CI]	Certain depression N (%)	OR [95%CI]
**Mechanical ventilation**										
**Yes**	15 (83.3)	1.7 [0.4-1,16]	5 (33.3)	4.5 [1.15-17.62]	15 (88.2)	4.54 [1.15-1.54]	10 (66.7)	3.21 [0.97-10.61]	7 (46.7)	2.87 [0.88-9.33]
**No**	51 (89.5)		6 (10)		45 (62.5)		23 (38.3)		14 (23.3)	
**p**	0.44		**0.04**		**0.014**		**0.04**		0.11	
**Fatigue**										
**Yes**	-	-	10 (90.9)	2.5 [1.07-1.33]	58 (87.9)	25.37 [4.46-144.08]	32 (96.9)	8 [1.55-2.49]	20 (30.3)	3.47 [0.34-25.82]
**No**	-	-	1(1.5)		2 (22.2)		1 (2.3)		1 (11.1)	
**p**	-		0.34		**<0.001**		**0.004**		0.43	
**Cognitive plaints**										
**Yes**	58 (96.7)	25.37 [4.46-144.08]	10 (16.7)	2.8 [0.33-23.78]	-	-	32 (96.9)	25.37 [1.68-2.94]	20 (33.3)	7 [0.85-57.08]
**No**	8 (53.3)		1 (6.7)		-	-	1(2.3)		1 (6.7)	
**p**	**<0.001**		0.45		-	-	**<0.001**		0.053	
**Very severe dyspnea**										
**Yes**	11 (78.6)		-	-	10(90.9%)	2.8 [0.33-23.78]	8 (72.7)	4.16 [1.01-17.18]	7 (63.6)	6.25 [1.59-24.49]
**No**	55 (90.2)	2.5 [0.5-1.28]	-	-	50 (78.1%)		25 (39.1)		14 (21.9)	
**p**	0.35		-	-	0.45		0.051		**0.009**	
**Certain anxiety**										
**Yes**	33 (97.1)	8 [1.08-1.49]	8 (24.2)	4.16 [1.01-17.19]	32 (96.9%)	25.37 [1.24-1.94]	-	-	19 (57.6)	27.14 [5.59-131.65]
**No**	33 (80.5%)		3 (7.1)		28 (59.5%)		-	-	2 (4.8)	
**p**	**0.035**		0.051		**<0.001**		-	-	**<0.001**	
**Certain depression**										
**Yes**	20 (95.2)	3.47 [0.41-29.68]	7 (33.3)	6.25 [1.59-24.45]	20 (95.2%)	7 [0.85-57.08]	19 (90.5)	27.14 [5.59-131.65]	-	-
**No**	46 (85.2)		4 (7.4)		40 (74.1%)		14 (25.9)		-	-
**p**	0.43		**0.009**		0.053		**<0.001**		-	-

OR: Odds Ratio; p: p value; CI: Confidence Interval

## Discussion

We found that the post-COVID symptoms most frequently cited by participants were fatigue, stress, and cognitive complaints. These symptoms affected functioning and activities, with 10.7% of patients having severe difficulties in carrying out domestic activities, 9.3% experiencing severe obstacles in carrying out social activities, and 8% facing severe challenges in performing their main occupations. This was also supported by Martina Betshart et al, who showed that most of the population perceived moderate to severe limitations affecting their ability to carry out their usual activities, and their quality of life [11]. By exploring the key concepts of fatigue, cognitive complaints, anxiety/depression, and dyspnea using validated scales, we were able to assess the degree of these symptoms. For the first concept (fatigue), based on the case definition of CFQ-11, 88% met the criteria for fatigue. Comparing the results of our study with other studies, we found that our result is more alarming than those of a study by Townsend *et al*. (2020), who pointed out that half of their sample met the criteria for fatigue (52.3%) [17], and a study in Saudia Arabia which (52%) of the participants were categorized as fatigued [18].

The analytical study of the factors determining fatigue showed that fatigue was more frequent in those who presented respiratory symptoms such as cough during the acute phase of COVID-19, those with pathological cognitive complaints, and those with definite anxiety. A study carried out in 2022 in Barcelona, Spain reinforced the connection between fatigue, cognitive complaints, and anxiety in patients who had experienced COVID-19 [19]. Furthermore, the cough experienced during the acute phase may be a sign of prolonged inflammation in the respiratory tract, which can continue to damage the respiratory system even after the resolution of the infection contributing to fatigue [20]. However, no significant difference was found between fatigue and gender, which contrasts with the study conducted in Egypt, which showed a significant difference between gender and fatigue [21]. Similarly, our study did not demonstrate a substantial difference between fatigue, geographical origin, and tobacco consumption, which was consistent with the findings of the same study conducted in Egypt [21].

### Cognitive complaints

We found that 80% of the participants had a score indicating a cognitive complaint. Of these, 82% were aware of their symptoms, as they had stated. This result was consistent with the findings of Ed Yong in 2022 which estimated that 65% to 85% of patients with long COVID had cognitive impairment [22], but it was higher than what was found in another study, which found clinically significant cognitive complaints were present in only 51% to 58% of patients [23]. The analysis of the factors influencing cognitive complaints showed that cognitive complaints were more frequent in those experiencing anxiety and fatigue. This finding is supported by the study conducted by Cavaco *et al*.[24]. Additionally, cognitive complaints were more frequent among those who underwent mechanical ventilation. This could be linked to the prolonged stay in intensive care where sedation and drugs were used, as well as the heavy weaning process from ventilation, which can retard recovery and lead to cognitive difficulties.

### Dyspnea

Several levels of dyspnea were determined based on the mMRC scale, representing the third part of our study. The prevalence of very severe dyspnea was 14.7%. This prevalence was high compared to a survey conducted in India in 2020, which showed a prevalence of severe dyspnea of 0.5% [25]. A significant difference was found between very severe dyspnea and those who were under mechanical ventilation. However, the current study's findings do not support the previous research conducted in Vancouver, Canada, which showed no significant difference in the severity of dyspnea compared to patients who did and did not require mechanical ventilation [26]. Plus, very severe dyspnea was more frequent in those aged between 70 and 95 years in comparison to those aged between 18 and 45 years with a significant association, a possible explanation for this might be the physiological decline in respiratory function with age. In addition, dyspnea was more frequent among individuals with depression, as revealed by a study conducted in the UK, which demonstrated a link between dyspnea and prior depressive conditions [27].

### Anxiety and depression

Our study shows a prevalence of anxiety and depression of 44% and 28% respectively. The prevalence of anxiety was similar to what was found in a study conducted in Turkey with a prevalence of anxiety of 46.5% but the prevalence of depression found in our study was lower than the same study (28% vs 36.1%) [6]. However, our study found a higher rate of depression and anxiety compared to a study conducted in Tunisia in 2021, which reported prevalences of 11% and 24.7% for depression and anxiety, respectively [28]. The analysis of factors associated with depression revealed that it was more common among non-workers. One possible explanation is that non-workers might have fewer regular social contacts. Work often provides a daily structure, personal goals structure, and social interaction, which can help maintain mental health. Plus, Anxiety levels were shown to be higher among people who were depressed. The fact that COVID-19 can affect the brain and the neurobiological systems that regulate emotions may assist in explaining this. Disrupting these systems may increase the risk of anxiety and depression [29]. In general, pathological psychiatric issues observed after the COVID-19 infection might be intensified by fear of death or serious illness, concerns about spreading the virus to others, the effects of social isolation, and the inflammatory and immune responses triggered by the virus [6]. This observation underscores the importance of considering mental health as a key element in assessing the long-term impacts of COVID-19.

### Strengths and limitations of the study

To the best of our knowledge, this is the first study to integrate the following four concepts: fatigue, cognitive complaints, dyspnea, anxiety, and depression. Additionally, the scales used to assess these four key concepts are validated. Nevertheless, our study was also limited by its cross-sectional design, which typically limits the confirmation of temporality and causality of factors.

## Conclusion

Our study highlights the most frequently observed post-COVID-19 symptoms. Our results revealed an alarming prevalence of symptoms with an overwhelming majority of patients showing signs of fatigue, cognitive complaints, anxiety, and depression. Dyspnea, although less frequent, is a major handicap for some patients. The analysis of the results showed that the level of cognitive complaint fatigue, dyspnea, anxiety, and depression varies from one subject to another and can be influenced by several socio-demographic factors such as gender, age, origin, or clinical factors such as medical history or symptoms presented during the acute phase of COVID such as cough. The challenge of this situation stems from the variety of symptoms and their effects on quality of life. Patients experience intense symptoms without a clear understanding of their origins. Consequently, it is crucial to focus on supporting these individuals to ease their symptoms and to promote awareness and guidance about post-COVID-19 syndrome.

### What is known about this topic

- The prevalence of post-COVID syndrome is considerable;
- Long-term impacts of COVID-19: a serious handicap in patients life;
- Fatigue, cognitive complaints, dyspnea, anxiety, and depression are the most frequently encountered symptoms in post-COVID syndrome.

### What this study adds

- Study of the four aspects of post-COVID-19 syndrome;
- High prevalences of post-COVID-19 symptoms;
- Fatigue is frequently encountered in those with cognitive complaints post-COVID.
